# Comparative analysis of contraceptive use in Punjab and Manipur: exploring beyond women’s education and empowerment

**DOI:** 10.1186/s12889-022-13147-3

**Published:** 2022-04-18

**Authors:** Anjali Sharma, Abhishek Kumar, S. K. Mohanty, Arupendra Mozumdar

**Affiliations:** 1 International Institute for Population Sciences (IIPS), Mumbai, India; 2 Population Council, New Delhi, India

**Keywords:** Modern contraceptive use, Women education, Women empowerment, FLWs outreach for family planning, Facility readiness, Multilevel analysis, India

## Abstract

**Background:**

Women’s education and empowerment are important predictors of contraceptive use across countries. However, two of the Indian states, namely, Punjab and Manipur, showed large variations in contraceptive use, despite the similar level of women’s educational attainment and empowerment. Therefore, this paper attempts to understand variation in contraceptive use between these states, despite having similar level of educational attainment and empowerment among the married women.

**Methods:**

This study primarily used cross-sectional data of the National Family Health Survey (NFHS) 2015–16 and to some extent the District Level Household Survey (DLHS) 2012–13 data. The analytical sample includes 13,730 currently married women in Punjab and 8,872 in Manipur. Modern contraceptive prevalence rate (mCPR) is the key outcome variable of this study. Bivariate, multivariate, and multilevel regression analysis are applied to understand the differences in mCPR between these states and its determinants.

**Results:**

Mean years of schooling was about 8 years among women of both the states, and about 34% of the women in Punjab and 27% of the women in Manipur have high level of autonomy. Despite this, use of modern method was 66% in Punjab and only 13% in Manipur. Coverage of family planning program indicators were significantly lower in Manipur than Punjab – frontline workers’ (FLWs) outreach for family planning was only 18% in Manipur compared to 52% in Punjab. Similarly, only 11% of the public health facilities in Manipur compared to 50% of the health facilities in Punjab were ready to provide at least one clinical method of family planning.

**Conclusion:**

Despite the similar level of individual level characteristics across the two states, poor coverage of family planning programs – low outreach of FLWs, low level of facility readiness, as well as sociocultural norms discouraging contraceptive use – might be responsible for lower contraceptive use in Manipur than Punjab. This implies for strengthening the health system for family planning in Manipur to meet the contraception needs of women by addressing sociocultural barriers in the state.

**Supplementary Information:**

The online version contains supplementary material available at 10.1186/s12889-022-13147-3.

## Background

India was the first country in the world to adopt government sponsored family planning program in 1952. Since then, family planning remained an integral part to national population policy and reproductive and child health program adopted and implemented in the country time to time. The various programs implemented after 1950s, intended to increase uptake of family planning services. Due to the longstanding efforts, use of contraceptive prevalence rate (CPR) has increased five folds over the past five decades in India – the CPR increased from 11% in 1970 to 54% in 2016 [1, 2], and due to the provision of voluntary choice in family size norm, there is decline from around six births per women in 1970 to about two births in recent years [3].

Previous research has identified women’s education and empowerment as important determinants of contraceptive use [4–15]. Women’s education enhances their status within and outside the family and increases exposure to the information and ideas disseminated through printed materials related to contraceptives. Some studies also argued that improved status of women increase their economic opportunities which further lead to higher decision-making power, hence higher control over contraceptive use [6, 16]. Increased education and empowerment among women also encourage inter-spousal communication on family planning which is conducive to promote contraceptive use among couples [17]. Despite these findings cutting across countries, evidence from India shows inter-state gap in level of contraceptive use, even when the women education and empowerment level are at similar in those states, which requires further exploration.

Previous studies have also focused that apart to the individual factors, family planning programs such as health worker outreach for family planning, improved supplies of family planning services, facility readiness to provide contraceptive methods also play important role in uptake of contraceptive use [17–19]. Health workers outreach for family planning is associated with generating demand for contraceptives, increased modern contraceptive use, lowering unmet need, and reducing overall fertility across many developing countries. Health workers’ frequent contact with women of the community, counselling/advising them for family planning use, and door to door distribution of contraceptives are key to success of family planning program. Addition to the health workers engagement, easy service provision and improvement in supply side activities for family planning is important for an increased and sustained use of contraceptives even with low levels of socio-economic development [17]. Addition to the individual and family planning program factors, cultural and societal norms such as religious barriers, misconception about side-effects, social stigma, son-preference, belief of proving fertility soon after marriage also plays vital role in contraceptive use [20].

Like many other developmental indicators, Indian states exhibit large inter-state variation in contraceptive use. Importantly, contraceptive use differs even among those states, where proximate as well as distal determinants of contraceptive use are similar. Punjab and Manipur are the two such states. However, in these two states, level of women education and empowerment – determining factors for contraceptive uses and fertility – are similar. This difference in contraceptive use, despite having similar educational and empowerment level is puzzling. If women’s education and empowerment is associated with contraceptive use, then why contraceptive use differs between the two states? The large gap between contraceptive use in Punjab and Manipur needs special attention as none of the literatures have explained this differential. Exploring the contraceptive use gap between the two states may help to unpack regional gap in contraceptive use and hence to adopt region specific family planning programming. In this context, this paper tries to explore the difference in modern contraceptive use between Punjab and Manipur, looking beyond women education and empowerment status.

## Materials and methods

### Data

This study primarily analysed fourth round of the National Family Health Survey (NFHS) data, conducted in India during 2015–16. The NFHS 2015–16 was conducted on representative samples of households covering all states and union territories of India. It aims to provide national and state level estimates on fertility, use of family planning, maternal and child healthcare services, childhood mortality, health and nutritional status of mother and their new-borns, knowledge and prevalence of HIV, among others. The survey also provides district level estimates for some of the indicators.

The NFHS adopted a multistage sampling design – a two-stage sampling design in rural areas and a three-stage design in most of its urban areas. In the survey, the information was collected from a nationally representative sample of 601,509 households and 699,686 women aged 15–49 years. The data was collected using household schedule and eligible women/individual schedule. The household response rate was 98% and the individual (women) response rate was 97%. In the state of Punjab, data was collected from state representative sample of 16,449 households, and 19,484 women aged 15–49 years; whereas in Manipur data was collected from a sample of 11,724 households, and 13,593 women aged 15–49 years. In both the states, the household response rate was over 98% and the individual (women) response rate was over 97% [2].

The main analysis of this study is supplemented by using the District Level Household Survey (DLHS) 2012–13 data. The DLHS is also a nationwide survey which aims to provide estimates of reproductive and maternal health indicators at district level in the country. Other than the community level information, the DLHS 2012–13 also collected information about availability of human resource, service provision etc. district hospitals (DH) and community health centres (CHC) across the country using facility survey schedule. Detail of the DLHS, report, and data is available at http://www.iipsindia.org.

### Measures

#### Outcome variable

The outcome variable of this study is modern contraceptive prevalence rate (mCPR) which is defined as proportion of currently married women aged 15–49 years using any modern contraceptive methods at the time of survey. This outcome was estimated based on two questions asked in the survey. First, currently married women were asked *‘Are you currently doing something or using any method to delay or avoid getting pregnant?’* Those who responded ‘yes’ were further asked ‘Which method are you using?’ Women who responded that they/their husbands were using female sterilization, male sterilization, an intrauterine contraceptive device (IUCD), male/female condoms, oral contraceptive pills, injectables or diaphragm were considered as using any modern contraceptive.

#### Key predictors

The key predictors in this study are women education and empowerment. Addition to these, family planning program variables were also used as predictors in the study. The predictors are defined as follows:

#### Women education

Women’s education level is computed based on completed years of schooling and grouped into four categories – no schooling, < 10 years of schooling, 10–12 years of schooling, and 12 + years of schooling.

#### Women empowerment

Women empowerment is composite indicator, which is computed using a set of standard variables, suggested in previous research. Previous studies have measured women empowerment based on indicators of women’s mobility (freedom to visit places unescorted) and decision-making [21, 22]. The NFHS 2015–16 provides sufficient information on all these indicators to compute a women empowerment index. Five decision-making indicators are used: (i) decision on own health care, (ii) decision on large household purchases, (iii) decision on visits to family and relatives, (iv) decision on spending husband’s earnings, (v) wife beating is justified if she refuses for sex. Four mobility indicators are used: (i) allowed to go to market, (ii) allowed to go to a health facility, and (iii) allowed to go outside the village (iv) beating justified if went outside without telling. Five economic indicators are used: (i) land ownership (ii) house ownership (iii) working status (iv) having bank account (v) having mobile phone. Using these indicators, a composite index is computed by applying Principal Component Analysis and termed as women empowerment index which is further divided into three categories: low, medium and high autonomy.

#### Family planning program related predictors

Family planning program coverage such as (i) health workers outreach for family planning, (ii) knowledge of place from where one can get contraceptives, (iii) Method Information Index (MII), (iv) exposure to family planning messages through media, (v) facility readiness for at least one clinical method are other key predictors. In the survey, information on first two FP program variables is collected from women who were not using any contraceptives at the time of survey. Method Information Index (MII) is a composite measure of quality of care and depicts the extent to which women were given specific information when they received family planning services from a facility/provider [23, 24]. The MII is computed using three questions asked to a modern method user – were you informed about other methods? Were you informed about side-effects of the method you adopted? Were you told what to do if you experienced side-effects? Those women who responded “yes” to all the three questions were considered under method information index. In this study the MII is calculated for women who were using a modern method of contraceptive. In the survey, all women were asked that whether they were exposed to FP messages through radio, television, newspaper, wall painting/hoarding. Those women who reported ‘yes' to either of the media channels were considered as exposed to FP messages.

Facility readiness for at least one clinical method is calculated using the DLHS (facility data) 2012–13 data. The readiness is calculated for facilities up to primary health centres and above. The readiness is defined if the facilities have required infrastructure, equipment, trained staff for providing at least one clinical method (female sterilization or intra uterine contraceptive device) and availability of either oral pills or condoms at the date of survey/observation.

#### Confounding variables

To assess the effect of aforesaid predictors on explaining the gap in modern contraceptive use in between the states, following variables are adjusted in the analysis: age of the women (15–24 years, 25–34 years, 35–49 years), number of living children/parity (0 child, 1 child, 2 children, 3 + children), current working status (no, yes), place of residence (rural, urban), household wealth index (poor, middle, rich), caste (Scheduled Caste [SC], Scheduled Tribes [ST], Other Backward Caste [OBC], Others), religion (Hindu, Non-Hindu), desire for additional children (no, yes), migration status of husband (no, yes). All these variables were found to be associated with contraceptive use in previous studies conducted in India and other developing countries [17–19, 25–34].

### Statistical analysis

Bivariate analysis was used to understand the socio-demographic profile of currently married women across the two states. The analysis is also applied to understand the differences in prevalence of modern contraceptives by selected characteristics as well as differences in coverage of family planning program indicators across the two states. To examine the factors associated with use of modern contraceptive, multivariate analysis is applied. Since outcome variable is dichotomous (for instance, 1 = using a modern method; 0 = otherwise) binary logistic regression analysis is used. In the regression analysis two models were run separately for Punjab and Manipur. In the model I, we only included women education and empowerment status, while in the model II, we included all the selected demographic and socioeconomic variables. Results obtained from the regression analysis are presented as odds ratio with 95% confidence intervals and corresponding significance level. In the regression analysis, the explanatory variables were tested for possible multi-collinearity – using Variation Inflation Factor (VIF) test. Pearson correlation analysis is applied to understand the association between FP program variables and modern contraceptive prevalence. Given that the information on some of the program variables is available to non-user women only, the correlation analysis was carried out by using district level average value of the program variables and mCPR.

The Pearson correlation analysis is not adjusted for other confounders, hence we further used multilevel analysis. In the multilevel analysis, individual level, household level, community level, and district level factors are considered together (details of the variables considered at different level was presented in Additional file 1: Appendix 1). The variable considered at household level was collapsed at household level indicating that those household with similar characteristics are considered as similar type of household. The primary sampling unit (PSU) of the NFHS generally coincides with villages in rural areas and census enumeration blocks in urban areas. The PSUs are a cluster of households and share a common geographical, ecological, and cultural environment which is considered as communities in this paper. Therefore, the variables measured as community/PSU are collapsed at the PSU level. The district level variables are mostly family planning program related variables and were generated from the individual level data by collapsing those at district level. The facility readiness variable was taken from the DLHS 2012–13 facility level data and merged with the NFHS data using district code. Considering these four levels of variables, multilevel regression model is applied to evaluate relationships between use of modern contraceptive and explanatory variables and their variances at different levels. In the multilevel analysis, we run three separate models. Model-I is empty model – without any explanatory variables – and fitted to test the random variability in the intercept. Model-II examined the effect of individual & household, and community level characteristics. Model-III examined the effect of individual & household, community, and district level characteristics simultaneously.

The fixed effects of explanatory variables were reported in terms of adjusted odds ratios (AORs) with their 95% confidence interval (CI). The random effects were expressed in terms of variances and standard errors at the household, community, and district levels. The random effects estimate the variation in use of modern method across different groups expressed as Intra-Class Correlation (ICC) and Proportional Change in Variance (PCV). We followed the similar procedures to fit multilevel analysis and compute ICC and VPC as suggested in previous studies [35, 36]. The estimates of binary logistic regression analysis as well multilevel analysis were presented as adjusted odds ratios (AORs) with 95% confidence intervals and corresponding significance level. AOR > 1 indicates higher odds of modern contraceptive use, while AOR < 1 indicates low use of modern contraceptive. All the statistical analyses are conducted using statistical software Stata 15.1. The multilevel analyses are carried out using the “runmlwin” program to run the MLwiN 3.00 beta software inbuilt in Stata. It is important to mention that the NFHS used multistage sampling design, hence standard errors were adjusted for weighting and clustering in all estimates.

## Results

### Socioeconomic characteristics of the study sample across the states

Percentage distribution of women by age group was similar across both the states and about half of the women across the states (49% each in Punjab and Manipur) were over 35 years of age (Table 1). Proportion of women with three and more children was 26% in Punjab and 38% Manipur. Desire for additional child was higher among women of Manipur (34%) than Punjab (20%). Mean years of schooling was about 8 years in both the states. In Punjab 39% of women have low autonomy, 27% have medium autonomy, and 34% have high autonomy. This compared with 28%, 45%, and 27% respectively in Manipur. Distribution of women by current working status was higher in Manipur (54%) than Punjab (18%). Women distribution by household wealth index varies across the states – 86% women of Punjab compared to only 31% women of Manipur belonged to rich household. Majority of women across the states were non-Hindu. In both the states, about 4% of women reported that their husbands were migrant.

**Table 1:** Percentage distribution of currently married women 15–49 years by selected demographic and socioeconomic characteristics in Punjab and Manipur, 2015–16

	**Punjab**	**Manipur**
**Age of women (in years)**		
15–24 years	11.0	13.5
25–34 years	40.4	37.6
35–49 years	48.6	49.0
* Mean age (in years)*	*34.4*	*34.1*
**Parity**		
0 child	8.6	9.1
1 child	22.2	23.6
2 children	42.8	29.0
3 + children	26.4	38.3
**Desire for more children**		
No	79.7	66.3
Yes	20.3	33.7
**Years of schooling**		
No schooling	21.5	15.3
< 10 years	48.6	56.4
10–12 years	15.0	14.5
12 + years	14.9	15.7
* Mean years of schooling*	*8.0*	*8.4*
**Women autonomy**		
Low	38.9	28.1
Medium	26.8	44.9
High	34.3	27.0
**Currently working**		
No	82.0	46.5
Yes	18.0	53.5
**Place of residence**		
Urban	40.1	38.5
Rural	59.9	61.5
**Household wealth index**		
Poor	3.5	37.7
Middle	10.8	31.3
Rich	85.7	31.0
**Religion**		
Hindu	36.1	47.3
Non-Hindu	63.9	52.7
**Caste**		
Scheduled caste	37.2	7.2
Scheduled tribes	0.2	30.4
Other backward class	20.0	18.2
Others	42.7	44.2
**Husband migration status**		
Living together	95.7	95.5
Staying elsewhere	4.3	4.5
**Total number**^a^ **of currently married women (15–49 years)**	**13,730**	**8,872**

^a^Numbers are based on unweighted cases

### Differences in modern contraceptive use across the states

Prevalence of modern contraceptive use varied starkly in Punjab and Manipur – use of any contraceptive was 76% in Punjab and 24% in Manipur; and use of modern contraceptive was 66% in Punjab and only 13% in Manipur (Table 2). Among the contraceptive users, female sterilization (38%) was the dominant method in Punjab followed by condom (19%) and traditional method (10%); whereas in Manipur, traditional method was the main method (11%) followed by oral pills and IUCD (4% of each). About a third of the currently married women (30%) of Manipur has unmet need for contraception, which was only 6% in Punjab.

**Table 2:** Percent of family planning indicators among currently married women aged 15–49 years in Punjab and Manipur, 2015–16

	**Punjab**	**Manipur**
**Use of any method (CPR)**	75.8	23.6
**Use of modern method (mCPR)**	66.3	12.7
**Method mix among users of any method**		
Female sterilisation	37.5	3.1
Male sterilisation	0.6	0.1
Oral pill	2.5	4.2
IUCD	6.8	3.7
Condom	18.9	1.4
Any traditional method	9.5	10.9
* Rhythm*	*6.2*	*1.6*
* Withdrawal*	*3.3*	*9.3*
Other Method	0.1	0.2
**Women with unmet need for contraception**	**6.2**	**30.1**

In both the states, modern contraceptive prevalence rate was higher among older women (Table 3). For instance, in Punjab, the prevalence was 37% among women aged 15–24 years, 63% among women 25–34 years and 76% among women 35–49 years. Similarly, in Manipur, the prevalence was 7% among women 15–24 years, 15% among women aged 25–34 years and 13% among women 35–49 years. In both the states, use of modern contraceptive increased by parity. Use of modern method was about twice higher among women who want no more children than women who want more children – 74% vs. 38% respectively in Punjab and 15% vs. 7% respectively in Manipur. In Punjab, modern contraceptive prevalence rate decreased with increased education level – 76% among uneducated women and 59% among those who received more than 12 years of schooling. In Punjab, use of modern method was 56% among women with low autonomy, 70% among women with medium autonomy, and 67% among women with high autonomy; the use was 10%, 14% and 15% respectively in Manipur.

**Table 3:** Variations in modern contraceptive prevalence rate (%) among currently married women aged 15–49 years by selected background characteristics in Punjab and Manipur, 2015–16

	**Punjab**	**Manipur**
**Age of women**		
15–24 years	36.9	6.8
25–34 years	63.4	14.8
35–49 years	75.5	12.7
**Parity**		
0 child	10.5	0.8
1 child	55.2	7.3
2 children	75.2	15.3
3 + children	79.6	16.9
**Desire for more children**		
No	73.7	15.4
Yes	37.6	7.3
**Years of schooling**		
No schooling	75.8	12.5
< 10 years	66.0	13.5
10–12 years	61.8	13.4
12 + years	58.5	9.2
**Women autonomy**		
Low	55.7	10.1
Medium	70.2	13.8
High	66.9	14.9
**Currently working**		
No	62.6	11.9
Yes	65.4	14.7
**Place of residence**		
Urban	65.3	12.9
Rural	67.1	12.6
**Household wealth index**		
Poor	68.1	12.6
Middle	70.4	13.6
Rich	65.8	11.9
**Religion**		
Hindu	64.6	12.8
Non-Hindu	67.3	12.6
**Caste**		
Scheduled Caste	68.0	12.9
Scheduled Tribes	44.7	11.4
Other Backward Class	64.5	12.0
Others	65.8	14.0
**Husband migration status**		
Living together	68.0	13.0
Staying elsewhere	30.4	6.4

### Factor associated with modern contraceptive use across the states

Logistic regression analysis was conducted separately in both states to examine the factors associated with modern contraceptive use. Results, without accounting for other factors (Model I), show that women education was inversely associated with use of modern contraceptive in both the states; however, the association was significant in Punjab only (Table 4). For instance, in Punjab, women who received 12 + years of schooling were 52% less likely (AOR = 0.52; CI = 0.35,0.76) to use modern contraceptive than women who received no years of schooling. Result was similar for women with other groups of schooling. Women empowerment has positive and significant influence on modern contraceptive use across both the states – with compare to women with low empowerment, the women with high empowerment in Punjab were 70% more likely (AOR = 1.70; CI = 1.31, 2.20) to use modern contraceptive and the women in Manipur were 61% more likely (AOR = 1.61; CI = 1.07, 3.67) to use modern contraceptive. When women education and empowerment were adjusted for other socioeconomic characteristics (Model II), effect of education continued to be similar in Punjab. In Manipur, though women education had positive influence on modern contraceptive use, but it was not statistically significant. Effect of women empowerment continued to be same in both the states, but result was statistically significant in Punjab only. In Punjab, compared to women with low empowerment, women with high empowerment were 45% more likely (AOR = 1.45; CI = 1.03, 1.94) to use modern contraceptive.

**Table 4:** Odds ratio (95% of confidence interval) – obtained from binary logistic regression analysis – showing the influence of demographic and socioeconomic characteristics on modern contraceptive use among currently married women aged 15–49 years in Punjab and Manipur, 2015–16

	**Punjab**				**Manipur**
	**Model I**	**Model II**	**Model I**		**Model II**
**Years of schooling**					
No schooling (Ref.)					
< 10 years	0.67 (0.49, 0.91)**	0.74 (0.53, 1.05)	1.19 (0.65, 2.17)	1.39 (0.72, 2.70)	
10–12 years	0.44 (0., 0.64)***	0.64 (0.40, 1.03)**	0.87 (0.41, 1.82)	1.13 (0.47, 2.73)	
12 + years	0.52 (0.35, 0.76)***	0.87 (0.53, 1.40)**	0.85 (0.40, 1.81)	1.16 (0.47, 2.88)	
**Women autonomy**					
Low (Ref.)					
Medium	1.88 (1.41, 2.50)**	1.77 (1.29, 2.42)**	1.45 (0.90, 2.34)	1.26 (0.74, 2.14)	
High	1.70 (1.31, 2.20)***	1.45 (1.03, 1.94)**	1.61 (1.07, 3.67)**	1.28 (0.73, 2.25)	
**Age of women**					
15–24 years (Ref.)					
25–34 years		1.92 (1.28, 2.87)**		2.03 (0.79, 5.19)	
35–49 years		1.69 (1.09, 2.64)**		1.21 (0.47, 3.16)	
**Parity**					
0–1 child (Ref.)					
2 children		3.29 (2.34, 4.64)***		4.28 (2.30, 7.19)***	
3 + children		2.62 (1.73, 3.96)***		4.70 (2.36, 9.35)***	
**Desire for more children**					
No (Ref.)					
Yes		0.59 (0.41, 0.84)**		0.89 (0.52, 1.51)	
**Currently working**					
No (Ref.)					
Yes		0.81 (0.59, 1.13)		1.08 (0.71, 1.66)	
**Place of residence**					
Urban (Ref.)					
Rural		1.27 (0.96, 1.68)**		0.96 (0.62, 1.48)	
**Household wealth index**					
Poor (Ref.)					
Middle		1.26 (0.57, 2.76)		1.60 (0.98, 2.61)*	
Rich		0.97 (0.46, 2.06)		1.22 (0.67, 2.23)	
**Religion**					
Hindu (Ref.)					
Non-Hindu		0.99 (0.76, 1.29)		1.19 (0.71, 1.99)	
**Caste**					
Scheduled Caste (Ref.)					
Scheduled Tribes		1.00		0.40 (0.18, 0.89)**	
Other Backward Class		0.79 (0.57, 1.10)		0.49 (0.22, 1.07)*	
Others		1.06 (0.78, 1.45)		0.79 (0.39, 1.59)	
**Husband migration status**					
Living together (Ref.)					
Staying elsewhere		0.19 (0.12, 0.30)***		0.38 (0.13, 1.11)*	

Ref.: Reference category

Figures in parenthesis are 95% confidence intervals; ***p<0.001; **p<0.05; *p<0.10

Other than women education and empowerment, women parity was positively and significantly associated with modern contraceptive use in both the states. Modern contraceptive use was significantly lower among women with a migrant husband compared to women whose husbands were staying with them – in Punjab women with migrant husbands were 19% (AOR = 0.19; CI = 0.12, 0.30) less likely to use modern contraceptive and the women in Manipur were 38% less likely (AOR = 0.38; CI = 0.13, 1.11) to use modern contraceptive.

### Differences in family planning program between the states

Coverage of family planning program indicators were significantly higher in Punjab than Manipur (Table 5). For instance, method information index was 34% in Punjab and 20% in Manipur. Similarly, health workers’ outreach for family planning (among non-user women) was 52% in Punjab and only 18% in Manipur. When comparing the reasons for non-use among women who were not using any method at the time of survey, 19% of the women in Manipur compared to 9% in Punjab reported opposition from family (mostly from husband) as a reason for non-use. Health related reason for not using a method was much higher in Manipur (30%) than Punjab (2%). There is high and positive correlation between modern contraceptive use and method information index (correlation coefficient = 0.712) and health workers outreach (correlation coefficient = 0.788) in Manipur.

**Table 5:** Selected family planning program coverage (%) and its correlation with modern contraceptive use in Punjab and Manipur, 2015–16

	**Percentage**			**Correlation coefficient**	
	Punjab	Manipur	*p*-value^	Punjab	Manipur
Method information index (MII)	34.4	20.1	< 0.001	0.726*	0.712*
FLWs outreach for FP	51.8	17.5	< 0.001	0.476*	0.788*
Knowledge about source of FP services	78.2	64.4	< 0.001	0.397*	0.900*
Not received any FP message through media	11.8	26.2	< 0.001	0.146	–0.628
Reason for non-use					
*Any opposition*	9.2	18.6	< 0.001	0.147	–0.704*
*Health related reasons*	2.3	29.9	< 0.001	0.332	–0.455
Facilities (primary health centers and above) ready for at least one clinical method	50.3	10.8	< 0.001	na	na

^*p*-values are obtained using chi2 test

Correlation coefficients are obtained by taking the average district level value of the modern contraceptive use and selected family planning program coverage indicators

^*^p < 0.05

Multilevel logistic regression analysis shows that relationship between use of modern method with all individual and household characteristics, adjusting for community and district (Table 6). The results obtained from the analyses indicate that adjusted odds ratio of modern contraceptive use remained similar for individual-level characteristics even after adjusting for contextual variables from a higher level. In addition, district level variables such as facility readiness for at least one clinical method was strongly and positively associated with use of modern contraceptives. For instance, in case when 20–40% of the health facilities in districts are ready for at least one clinical family planning method, the adjusted odds ratio of modern contraceptive use was 1.68 [CI: 0.82, 3.47], and when over 40% of health facilities were ready, the adjusted odds ratio was 1.64 [CI: 0.73, 3.66] compared to when the < 20% of health facilities were ready for at least one clinical method of family planning. Another district level variable such as method information index was positively and significantly associated with use of modern contraceptive – adjusted odds ratio was 3.36 (CI: 1.65, 6.84] when the index was 40 + % compared when it was < 20%. Community level factor such as frontline health worker outreach was directly associated with use of modern contraceptive; however, the result was not significant statistically.

**Table 6:** Multilevel regression analysis showing associated factors to use of modern contraceptive (mCPR) among currently married women aged 15–49 years in Punjab and Manipur, 2015–16

	**Model-I**	**Model-II**	**Model-III**
	**Odds ratio (95% CI)**	**Odds ratio (95% CI)**	**Odds ratio (95% CI)**
**Individual & household level variables**			
**State**			
Punjab (Ref.)			
Manipur		0.05 (0.03, 0.08)***	0.12 (0.06, 0.24)***
**Years of schooling**			
No schooling (Ref.)			
< 10 years		1.03 (0.78, 1.36)	1.03 (0.78, 1.36)
10 + years		0.77 (0.56, 1.05)	0.77 (0.56, 1.05)
**Women autonomy**			
Low (Ref.)			
Medium		1.48 (1.17, 1.88)***	1.47 (1.16, 1.86)***
High		1.37 (1.08, 1.76)**	1.35 (1.06, 1.72)**
**Age of women (in years)**			
15–24 years (Ref.)			
25–34 years		2.10 (1.50, 2.95)***	2.10 (1.50, 2.94)***
35–49 years		1.79 (1.23, 2.60)**	1.77 (1.22, 2.57)**
**Parity**			
0–1 child (Ref.)			
2 children		3.10 (2.36, 4.09)***	3.05 (2.32, 4.00)***
3 + children		3.06 (2.21, 4.24)***	3.03 (2.19, 4.19)***
**Desire for more children**			
No (Ref.)			
Yes		0.58 (0.44, 0.77)***	0.57 (0.43, 0.76)***
**Currently working**			
No (Ref.)			
Yes		1.03 (0.81, 1.30)	1.03 (0.82, 1.31)
**Religion**			
Hindu (Ref.)			
Non-Hindu		0.91 (0.72, 1.14)	0.97 (0.77, 1.21)
**Caste**			
SC/ST (Ref.)			
OBC		0.86 (0.65, 1.13)	0.84 (0.64, 1.12)
Others		0.97 (0.75, 1.25)	0.94 (0.73, 1.21)
**Household wealth index**			
Poor (Ref.)			
Middle		1.18 (0.90, 1.55)	1.17 (0.89, 1.53)
Rich		1.26 (0.96, 1.65)	1.26 (0.96, 1.64)*
**Community level variables**			
**Place of residence**			
Rural (Ref.)			
Urban		0.88 (0.68, 1.14)	0.87 (0.67, 1.12)
**FLWs outreach for FP**			
< 30% (Ref.)			
30–60%		1.15 (0.86, 1.54)	1.12 (0.84, 1.50)
60 + %		1.27 (0.92, 1.74)	1.18 (0.86, 1.62)
**District level variables**			
**Method information index**			
< 20% (Ref.)			
20–40%			1.19 (0.66, 2.15)
40 + %			3.36 (1.65, 6.84)***
**Exposure to FP message through media**			
< 25% (Ref.)			
25 + %			0.91 (0.32, 2.58)
**Knowledge about source of FP services**			
< 20% (Ref.)			
20–40%			1.39 (0.92, 2.09)
40 + %			0.72 (0.32, 1.62)
**Facility readiness for at least one clinical method**			
< 20% (Ref.)			
20–40%			1.68 (0.82, 3.47)
40 + %			1.64 (0.73, 3.66)
**Constant**	0.85 (0.51, 1.41)	0.48 (0.27, 0.84)**	0.15 (0.05, 0.47)***
**Random effect parameters**			
Variance [SE]			
District level	1.90 [0.50]	0.26 [0.10]	0.05 [0.04]
Community level	0.19 [0.02]	0.43 [0.09]	0.43 [0.09]
Individual & household level	0.00 [0.00]	0.00 [0.00]	0.00 [0.00]
**VPC (PCV in %)**			
District level			0.01 (80.8%)
Community level			0.12 (0.0%)
Individual & household level			0.00 (0.0%)

*Ref.* Reference categories, *VPC* Variance partition coefficient, *PCV* Proportional change in variance, *CI* Confidence interval

^***^p < 0.001; **p < 0.05; *p < 0.10

*SE*: Standard errors; Figures in square brackets are standard errors

## Discussion

Using the National Family Health Survey 2015–16 and the District Level Household and Facility Survey 2012–13, this paper explores gap in modern contraceptive use among the currently married women aged 15–49 years between Punjab and Manipur, where women education and empowerment level are similar. Modern contraceptive prevalence rate differs starkly between these states – the mCPR is on highest end in Punjab and lowest end in Manipur. Women empowerment has a positive and significant influence on use of modern contraceptive in both the states. Women education has differential effect across the states – in Punjab it has significant negative effect, whereas in Manipur it has positive, but not significant, effect. Coverage of family planning health program variables is significantly lower in Manipur than Punjab.

Regional differences in use of family planning services are evidenced in previous studies and our findings are similar [37, 38]. The stark regional variation in contraceptive use can be explained by the fact of inter-state differences in women’s characteristics which determine contraceptive use, differences in family planning program coverage, differences in socioeconomic status of the states, as well as inherited cultural/social norms related to family planning use in the states. While in both the study states, some of the characteristics of women i.e. education level, empowerment etc., are similar, and hence raises the question that why there is huge differences in modern contraceptive use despite having similar level of characteristics.

Women empowerment has significant positive influence on use of modern contraceptive in Punjab but not in Manipur. This finding in case of Punjab is similar to that of previous studies which documented that empowered women are more likely to adopt family planning services [4, 5, 8–12, 14]. Role of high empowerment on modern contraceptive use can be channelized through greater knowledge and importance of family planning method, increased inter-spousal communication on family planning, increased economic opportunity among women, better decision making about their reproductive matters, and increased confidence on seeking family planning services with a health provider which all can affect contraceptive use in positive way. Our findings showed that women’s education has negative effect on modern contraceptive use, particularly in Punjab. In prosperous state like Punjab, other developmental indicators and FP program strengthening might be playing greater role in adoption of modern contraceptive than the women education.

The differences between coverage of family planning program indicators between the two states suggest that family planning program intensity are different in these two states and can be possibly associated with different level of modern contraceptive use between the states. A stark gap in informed choice between the states is associated with the gap in modern contraceptive use. For instance, the results obtained from multilevel analysis clearly indicate that facility readiness directly associated with use of modern method. Given the very low level of facility readiness in the state of Manipur than Punjab, that could be one of the reasons for such a low use of family planning services in the state. The findings further revealed that higher the method information index, higher the use of family planning. This finding is in similar line with previous studies, which found that quality of care leads to increased contraceptive uptake and continuation [39–42].

Health workers’ outreach for family planning is also higher in Punjab than in Manipur, which may contribute in contraceptive use gap between the states. Health workers’ frequent contact with women of the community, counselling/advising them for family planning use, and door to door distribution of contraceptive services has been associated with increased demand and use of the family planning services [43, 44]. High level of opposition and health related reasons for non-use in Manipur than Punjab might be other factors for contraceptive use differentials between the states. Previous studies have shown that side-effect or fear of side-effect is strongly associated with either choosing not to start or discontinuing contraceptive [45–47]. Results of the multilevel analysis showed that inclusion of the district level variables decrease the randomness in the model, which reflects that influence of district level variables in explaining the variation in contraceptive use.

Findings of this study needs to be interpreted cautiously considering few limitations. First, the study used cross-sectional survey data, which can only reveal association rather than causal effect between outcomes and covariates. Second, these two states are culturally different, hence one could expect differing cultural/social norms encouraging to contraceptive use; however, information to those issues are not captured in the data set hence not analysed. Third, there may be important unmeasured factors that explain the observed associations, which were not captured in the dataset used in this study. This indicates that there is need for further research at ground level through primary survey to understand the cultural implication on contraceptive use. Finally, while the state specific multilevel analysis could have better reflected that how the programmatic factors are associated with modern contraceptive use within in the state, however given the few numbers of districts, particularly in Manipur, we could not conduct the multilevel analysis sperate for the states.

## Conclusion

The findings of this study offer research as well policy implications in context of varying contraceptive use across geographies of India. First, there is scope of more work to explain contraceptive use gap between the two states. This can be done by conducting primary study to understand the historical and cultural perspective on contraceptive use across the states, particularly in Manipur. Qualitative information may be collecetd to understand the cultural barrier in use of family planning. Second, there is need to improve the family planning program coverage indicators such as increased provider client interaction, facility readiness to offer more basket of choice, counselling of women on fear of side-effect, family/community involvement on family planning discussion, and increased health workers outreach for family planning. All these will help in uptake of use of contraceptive services, particularly in Manipur and those states where prevalence of modern contraceptive is low.

## Supplementary Information

**Additional file 1:** **Appendix 1**. List of predictors at different level used in the multilevel analysis.

## Data Availability

The NFHS data can be downloaded from www.DHSprogram.com and the DLHS data can be obtained from http://www.iipsindia.org.

## Abbreviations

CHC: Community Health Centres
CI: Confidence Intervals
CPR: Contraceptive Prevalence Rate
DH: District Hospitals
DLHS: District Level Household Survey
FLW: Frontline workers
FP: Family Planning
mCPR: Modern Contraceptive Prevalence Rate
MII: Method Information Index
NFHS: National Family Health Survey
PSU: Primary Sampling Unit

## References

[1] IIPS (2014). District Level Household and Facility Survey (DLHS–4), 2012–13: India.

[2] IIPS (2017). ICF: National Family Health Survey (NFHS–4), India, 2015–16.

[3] Goli S, Arokiasamy P (2013). Demographic transition in India: an evolutionary interpretation of population and health trends using change-point analysis. PloS One.

[4] Jain A, Nag M (1986). Importance of female primary education for fertility reduction in India. Econ Pol Wkly.

[5] Singh SK, Sharma B, Vishwakarma D, Yadav G, Srivastava S, Maharana B (2019). Women's empowerment and use of contraception in India: macro and micro perspectives emerging from NFHS-4 (2015–16). Sex Reprod Health.

[6] Riyami AA, Afifi M (2003). Women empowerment and marital fertility in Oman. Egypt Public Health Assoc.

[7] Riyami AAI, Afifi M, Mabry RM (2004). Women's autonomy, education and employment in Oman and their influence on contraceptive use. Reprod Health Matters.

[8] Alabi O, Odimegwu CO, De-Wet N, Akinyemi JO (2019). Does female autonomy affect contraceptive use among women in northern Nigeria?. Afr J Reprod Health.

[9] Amraeni Y, Kamso S, Prasetyo SB, Ahmad M (2020). Women's involvement in decision making fo unmet need for contraception in Indonesia. Enferm Clin.

[10] Asaolu IO, Okafor CT, Ehiri JC, Dreifuss HM, Ehiri JE (2016). Association between measures of women's empowerment and use of modern contraceptives: an analysis of Nigeria's demographic and health surveys. Front Public Health.

[11] Blackstone SR (2017). Women's empowerment, household status and contraception use in Ghana. J Biosoc Sci.

[12] Crissman HP, Adanu RM, Harlow SD (2012). Women's sexual empowerment and contraceptive use in Ghana. Stud Fam Plann.

[13] Dhak B, Saggurti N, Ram F (2019). Contraceptive use and its effect on Indian women's empowerment: evidence from the national family health survey-4. J Biosoc Sci.

[14] Hameed W, Azmat SK, Ali M, Sheikh MI, Abbas G, Temmerman M, Avan BI (2014). Women’s empowerment and contraceptive use: the role of independent versus couples’ decision-making, from a lower middle income country perspective. PLoS One.

[15] Palamuleni ME, Adebowale AS (2014). Women empowerment and the current use of long acting and permanent contraceptive: evidence from 2010 Malawi demographic and health survey. Malawi Med J.

[16] Phan L (2013). Women's empowerment and fertility changes. Int J Sociol Fam.

[17] Mahmood N, Ringheim K (1996). Factors affecting contraceptive use in Pakistan. Pak Dev Rev.

[18] Raut M, Sekher T. Determinants of contraceptive use in Non-EAG and EAG states of India in the era NRHM: evidences from Gujarat and Odisha, 2009–10. J Fam Welfare. 2014;60(1):41–52.

[19] Palamuleni ME (2013). Socio-economic and demographic factors affecting contraceptive use in Malawi. Afr J Reprod Health.

[20] Najafi-Sharjabad F, Yaha S, Rahman A (2013). Barriers of modern contraceptive practice among Asian women: a mini literature review. Glob J Health Sci.

[21] Jejeebhoy SJ (1995). Women’s education, autonomy and reproductive behavior: Experience from Developing Countries.

[22] Saikia N, Singh A (2009). Does type of household affect maternal health? evidence from India. J Biosoc Sci.

[23] Jain AK (2017). Information about methods received by contraceptive users in India. J Biosoc Sci.

[24] Rana MdJ, Jain AK (2019). Do Indian women receive adequate information about contraception?. J Biosoc Sci.

[25] Thyagarajan S, Reji B, Viswan SP (2014). Determinants of contraceptive usage in India. Int J Interdiscip Multidiscip Studies.

[26] UNFPA (2012). A decade of change in contraceptive use in Ethiopia.

[27] Valekar S, Chawla P, Tukaram H, Fernandez K, Kalra K (2017). The socio-demographic determinants of contraceptive use among rural women in reproductive age group. J Women's Health Care.

[28] Lutalo T, Kidugavu M, Wawer MJ (2000). Trends and determinants of contraceptive use in Rakai district, Uganda, 1995–98. Stud Fam Plann.

[29] Dwivedi L, Ram F, Reshmi R (2007). An approach to understanding change in contraceptive behaviour in India. Genus.

[30] Chacko E (2001). Women's use of contraception in rural India: a village-level study of four villages in rural West Bengal. India Health and Place.

[31] Appiah F, Seidu AA, Ahinkorah BO, Baatiema L, Ameyaw EK (2020). Trends and determinants of contraceptive use among female adolescents in Ghana: analysis of 2003–2014 demographic and health surveys. SSM Population Health.

[32] Hiremath RN, Yadav AK, Ghodke S, Yadav J (2018). Contraceptive use and its determinants amongst armed forces personnel. Med J Armed Forces India.

[33] Majumder N, Ram F (2015). Contraceptive use among poor and non-poor in Asian countries: a comparative study. Soc Sci Spectrum.

[34] Arokiasamy P (2002). Gender preference, contraceptive use and fertility in India: regional and development influences. Int J Popul Geogr.

[35] Joseph KJV, Mozumdar A, Lhungdim H, Acharya R (2020). Quality of care in sterilization services at the public health facilities in India: a multilevel analysis. PLoS One.

[36] Ahmad J, Khan N, Mozumdar A (2019). Spousal violence against women in India: a social–ecological analysis using data from the national family health survey 2015 to 2016. J Interpers Violence.

[37] Wafula S (2015). Regional differences in unmet need for contraception in Kenya: insights from survey data. BMC Women's Health.

[38] Lakew Y, Reda A, Tamene H, Benedict S, Deribe K (2013). Geographical variation and factors influencing modern contraceptive use among married women in Ethiopia: evidence from a national population based survey. Reprod Health.

[39] Koenig MA, Hossain MB, Whittaker M (1991). The influence of quality of care upon contraceptive use in rural Bangladesh. Stud Fam Plann.

[40] RamaRao S, Lacuesta M, Costello M, Pangolibay B, Jones H (2003). The link between quality of care and contraceptive use. Int Fam Plan Perspect.

[41] Mensch B, Arends-Kuenning M, Jain A (1996). The impact of the quality of family planning services on contraceptive use in Peru. Stud Fam Plann.

[42] Tumlinson K, Okigbo C, Speizer I (2015). Provider barriers to family planning access in urban Kenya. Contraception.

[43] Mwaikambo I, Speizer IS, Schurmann A, Morgan G, Fikree F (2011). What works in family planning interventions: a systematic review?. Studies Fam Plann.

[44] Reifsnider E, Mendias EP, Davila YR (2012). Foreword: community health workers: full members of the health care. Fam Community Health.

[45] Casterline JB, Sathar ZA, Haque M (2001). Obstacles to contraceptive use in Pakistan: a study in Punjab. Stud Fam Plann.

[46] Machiyama K, Cleland J (2014). Unmet need for family planning in Ghana: the shifting contributions of lack of access and attitudinal resistance. Stud Fam Plann.

[47] Sedgh G, Hussain R (2014). Reasons for contraceptive non-use among women having unmet need for contraception in developing countries. Stud Fam Plann.

