# Supplementary material for: Comparative analysis of contraceptive use in Punjab and Manipur: exploring beyond women’s education and empowerment
Source: BMC Public Health. 2022 Apr 18;22:781. doi: 10.1186/s12889-022-13147-3 (PMC9016937; doi:10.1186/s12889-022-13147-3)
Supplement: Supplementary file 1 — Additional file 1: Appendix 1. List of predictors at different level used in the multilevel analysis. [file 12889_2022_13147_MOESM1_ESM.docx]

**Appendix 1** List of predictors at different level used in the multilevel analysis

| **Different level** | **Variables** | **Categories** |
| --- | --- | --- |
| Individual level | Women empowerment | Low, medium, high |
|  | Women education | Never attended school, 1-9 years of schooling, 10+ years of schooling |
|  | Age of women (in years) | 15-24 years, 25-34 years, 35-49 years |
|  | Parity of women | 0/1 child, 2 children, 3 and more children |
|  | Current working status | Not working, working |
|  | Desire for more children | No, yes |
|  | Caste | Scheduled caste/scheduled tribes [SC/ST], other backward castes [OBC], others |
|  | Religion | Hindu, non-Hindu |
| Household level | Household wealth index | Poor, middle, rich |
| Community/PSU level | Place of residence | Rural, urban |
|  | Frontline health workers outreach for family planning | <30%, 30-60%, 60+% |
| District level | Method information index | <20%, 20-40%,40+% |
|  | Facility readiness for at least one clinical method | <20%, 20-40%, 40+% |
|  | Exposure to FP message through media | <25%, 25+% |
|  | Knowledge about source of family planning services | <20%, 20-40%, 40+% |
